# Comparison of post-operative pain prevalence after single visit endodontic treatment with two NiTi rotary files - a randomized clinical trial

**DOI:** 10.1038/s41598-025-29029-8

**Published:** 2025-12-18

**Authors:** M. E. Khallaf, Yousra Aly, Amira Ibrahim Mohamed

**Affiliations:** Restorative and Dental Materials Department, National Research Centre, Dokki, Giza, Egypt

**Keywords:** Post-operative pain, ProTaper, M-Pro, Single visit, Diseases, Health care, Medical research

## Abstract

In root canal treatment, post-operative endodontic pain is considered as a common post-operative complication. Knowledge about its causes helps the clinician in proper instrument and technique selection to decrease its incidence. Therefore, the aim of this randomized clinical trial was to compare the post-operative pain occurrence after single visit root canal preparation using ProTaper Universal rotary system or M-Pro rotary system. Eighty patients with symptomatic irreversible pulpitis in mandibular first molars were allocated into two groups. In group A (*n* = 40): root canal preparation was performed using ProTaper Universal system and in group B (*n* = 40): root canal preparation was performed using M-Pro rotary system. Pain level was assessed by the patient using the numerical rating scale (NRS) at 24 h and 7 days postoperatively. The patients were advised to take the prescribed analgesics in case of emergency need. Data and statistical analysis showed that there was significant decrease in pain in both groups after 24 h and after 7 days. Insignificant difference was found between ProTaper Universal group and M-Pro group after 24 h and after 7 days. Insignificant difference was found in analgesics intake between both groups after 24 h and after 7 days.

Trial registration: The trial protocol was registered at https://ClinicalTrials.gov (NCT06777381), registered January 15, 2024.

## Introduction

Expecting pain during root canal treatment is considered the major cause of patient fear. Dealing with this issue is a major challenge for every dentist^1^. Post-operative endodontic pain had been defined as an unpleasant sensation caused by actual or potential tissue damage^2^. According to previous literature review, post endodontic pain was recorded to vary between 3 and 58%^3^. While preparing the root canal system, extrusion beyond the apical foramen of debris of the pulp and periapical tissues, microorganisms or dentin chips may contribute to postoperative pain^4^.

Giving that, instrument design and instrumentation techniques influence the amount of extruded debris apically, numerous studies have confirmed the ability of some rotary systems in reducing this extrusion^5,6^, leading to less pain sensation, especially when full sequence rotary instrumentation was used^7–9^. While other researchers concluded, that extrusion of some debris apically happens with all root canal instrumentation systems^10^.

This actually happens with both ProTaper Universal and M-Pro systems, but the amount varies based on many factors, such as file’s design, cross-sectional area, and its way of use^11^.

There is a controversy about the amount of debris extruded when M-Pro is used. Some studies showed that, M-Pro produces less debris than other systems; while other studies revealed that M-Pro produces the most apically extruded debris as it possess a large cross-section and less clearance ability^12,13^. Variations in results between studies may be attributed to differences in experimental factors^14,15^.

ProTaper Universal system (Dentsply Tulsa Dental Specialties, USA) is a well-known convex, triangular cross-section engine-driven multiple-file system with continuous rotational movement that was released in market in 2001^16^. ProTaper files are uniquely characterized by having a progressively tapered design; each instrument has changing percentage tapers over the length of its cutting blades. This special feature helps in improving flexibility, cutting efficiency and safety^17^.

In 2015, MPro (IMD, Shanghai, China) was introduced in the market. It is manufactured from the CM wire, which is characterized by containing a reduced percentage of nickel than other rotary systems^18^. This technology was developed to enable the instrument to have superior flexibility, providing the files the ability to preserve the original canal anatomy. It also enhances the safety with efficiency during root canal preparation. The canal shaping can be completed with continuous rotation motion and anti-screwing effect; associated with the possibility of pre-curving the file in inaccessible canal access achieving high cutting efficiency and appropriate debris removal with less debris extrusion^19^.

Therefore, the aim of this randomized clinical trial was to assess and compare the postoperative pain intensity in molars with symptomatic irreversible pulpitis after single endodontic treatment visit using either ProTaper or MPro rotary systems. The null hypothesis was that there would be no significant difference in postoperative pain intensity between ProTaper and MPro systems.

## Materials and methods

### Study design

A presented randomized clinical trial was performed at the National Research Centre dental clinic over a period of 4 months from October 2024 to the end of February 2025.

### Ethics

The Medical Research Ethics Committee, National Research Centre approved this randomized clinical trial; approval number (03460224).

### Sample size calculation

Sample size was calculated referring to Nekoofar et al.^20^. In accordance with this study, the minimally accepted sample size was 22 per group, when mean ± standard deviation of pain after 6 h in group I was 1.27 ± 2.3 while estimated mean difference was 2, estimated mean difference was calculated depending on the study conducted by Klukowska et al.^21^, when the power was 80% & type I error probability was 0.05. Total sample size increased to 27 per group to compensate for the 20% drop out. The t test was performed by using P.S. power 3.1.6.

### Selection of subjects

The subjects of the study were recruited from the endodontic clinic. A total of eighty adult patients were assigned into this study. Patients’ diagnosis was set to be symptomatic irreversible pulpitis in mandibular first molar. The diagnosis was established by endodontist based on chief complaint and clinical examination. Pulp vitality was confirmed with cold pulp test (Endo-Ice; Hygienic, USA) and an electric pulp test. The radiographic examination was done by intra-oral periapical film.

### Inclusion criteria

The assigned patients were males and females in a good health, their ages ranged from 25 to 45 years old, medically-free of any systemic disease, symptomatic irreversible pulpitis in lower first molar with mature roots, restorable teeth, and teeth without internal or external root resorption and teeth without calcified root canals.

### Exclusion criteria

Pregnant females, patients suffering from pain in multiple molars on the same side, subjects whose teeth demonstrated a positive response to percussion testing, and patients who had administered analgesics within 12 h prior to the initiation of the root canal treatment in this clinical trial were excluded. Additionally, teeth exhibiting grade 2 or 3 mobility, presence of periapical radiolucency, or teeth associated with extra oral or intraoral sinus tract or fistula were also excluded.

### Procedural steps

Patient Personal information includes the participant’s name, phone number, address medical and dental history was recorded. Chief complaint and history of pain were recorded in the patient’s own words.

### Randomization, subject allocation and blinding

Randomized sequences of integers ranging from 1 to 80 were established using RANDOM.ORG-Sequence Generator (http://www.random.org) and distributed into two lists on an Excel sheet representing two groups A and B, according to the rotary file system that would be used for root canal treatment. Each patient picked a number, and then the patient was allocated in either group according to this number. All procedures of root canal treatment were performed by two skilled operators (endodontists). The two operators were randomly assigned to the patients of each group so that each operator had an equal number of patients from both lists. This study is considered as single blind study (Subject Blinding), as the rotary file system that was used for root canal preparation was unrevealed to the patient.

### Clinical procedures

Before the start of this clinical trial, the operators agreed about a standardization protocol for each instrumentation system.

First, the preoperative pain for each patient was recorded using NRS, followed by tooth anesthesia by inferior alveolar nerve block injections using a carpule consisting of 2% mepivacaine hydrochloride with 1:100,000 epinephrine (Alexandria Company for pharmaceuticals and chemical industries). Access cavity was prepared with safe ended endo Z bur (Dentsply, Switzerland). Proper tooth isolation was done by rubber dam. Canal patency was checked by inserting a manual 10 K- file (Mani Inc., Japan) followed by pulp extirpation by 15 H-file (Mani Inc., Japan). Working length determination of the canal was established by electronic apex locator (Root ZX. Morita Corporation, Japan) and set to be1 mm shorter than the apex of the root. This reading was also approved by radiograph. Subjects were assigned into two groups: Group A: Root canal preparation was performed with ProTaper Universal system rotary files (Dentsply, Maillefer, Ballaigues, Switzerland). Group B: Root canal preparation was performed by M-Pro rotary files. All files were used to the full working length.

### In group A (ProTaper universal system)

Mechanical preparation was done as follows:

Starting by Shaping file (S1) introduced into the canal with a brushing movement, shorter by 3 mm of the estimated working length, then Shaping file (SX) with the same brushing motion was introduced into the canal by 2/3 of its blade length. Recapitulation was done by K file # 15 to the full working length. The full working length was reached by Shaping file (SI) followed by Shaping file (S2). Finishing file (F1) was used reaching the full working length, and the canal was then assessed with an ISO # 20 file. If it fits snuggly at the apex, the preparation was considered completed. If not, instrumentation was continued with the Finishing file (F2) and the canal was again reassessed with ISO # 25 file, if it fits snuggly at the apex, instrumentation was completed; if not, it was continued with Finishing file (F3).

### In group B (M-Pro rotary files)

Mechanical preparation was done as follows:

Flaring of coronal 2/3 of the canal was accomplished by the orifice opener (size 18 taper 9%). Canal preparation was proceeded by second file (size 20 taper 4%), then third file (size 25 taper 6%) and finally, the preparation was completed by the last file size (35 taper 4%). The three files were used to full working length, with in and out slow pecking motion.

In both groups, the rotary files were used with preprogrammed motor (X-Smart) with adjusted speed and torque according to the manufacturers’ instructions. In both groups, irrigation succeeding each file was achieved using 3 ml of 2.5% sodium hypochlorite (NaOCl). Final irrigation was performed with sterile saline and 3 ml of 17% Ethylenediaminetetraacetic acid (EDTA) solution for 1 min with a purpose of removing smear layer. Then, a final wash was performed by saline. A fitted suitable size Master cone was checked by radiograph. After canal dryness with suitable size paper points, root canal obturation was done using matched-size gutta-percha points and resin-based sealer ADSEAL (META BIOMED CO., LTD) by a modified single cone technique. Then, the pulp chamber was sealed with a well inserted cotton pellet followed by KETAC Silver Glass Ionomer Aplicap temporary restoration (3 M ESPS, Germany).

**Fig. 1:**
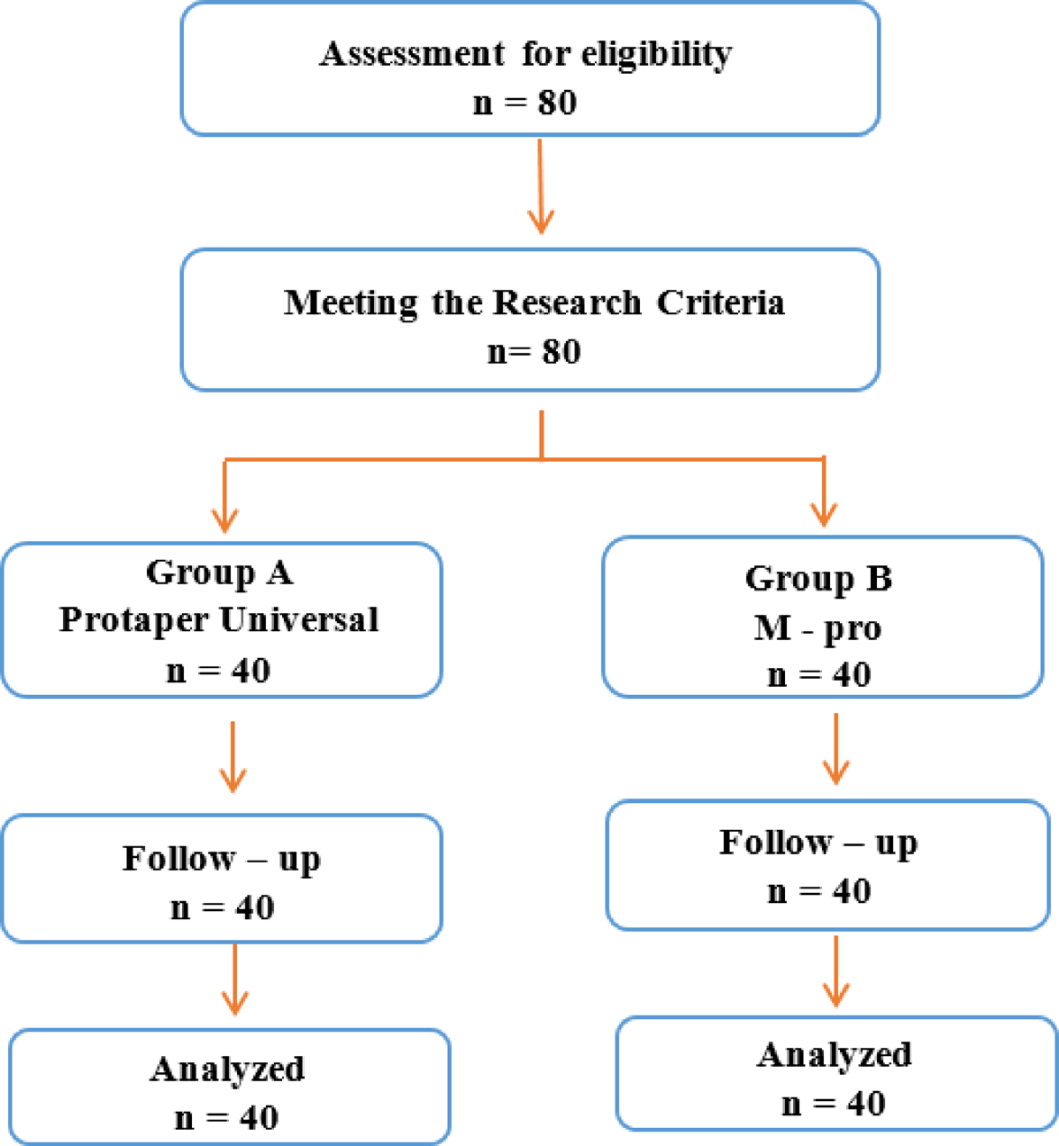
Flow Diagram showing the patients enrollment in the clinical trial and the main details of the study.

Patients were instructed about the right way to mark their pain intensity by using NRS after 24 h and 7 days postoperatively. Pain assessment was evaluated using a 10-point NRS, where the endpoints are the extremes of no pain and worst pain. The first operator explained to his patients’ group how to rate their pain sensation using NRS, and he also explained by the same way to most of the patients of the second operator. The severity of pain was assigned as follows: (0) equivalent to none/ (1) equivalent to slight pain / (2) equivalent to moderate pain and (3) equivalent to severe pain. Ibuprofen (200 mg) was prescribed in case of not tolerated pain^5^. This was illustrated in (Fig. 1).

## Results

### Inter-operator reliability evaluation

The Kappa values for all time points and groups indicate excellent inter-operator reliability in assessing pain.

In Group A, Kappa values were 0.923 at 24 h and 0.829 at 7 days, reflecting almost perfect agreement according to Landis and Koch’s classification (Kappa ≥ 0.81), while in Group B, Kappa values were 0.856 at 24 h and 0.780 at 7 days, showing substantial to almost perfect agreement (Kappa between 0.61 and 0.80 = substantial).

All results were statistically significant (*P* < 0.0001), confirming that the observed agreements were not due to chance, as presented in (Table 1).

**Table 1:** Reliability between 1st and 2nd operator regarding pain.

Kappa		Kappa	Asymptotic standard error	Approximate T	Approximate significance
Group A	24 h	0.923	0.075	6.649	< 0.0001*
	7 days	0.829	0.117	4.748	< 0.0001*
Group B	24 h	0.856	0.097	5.913	< 0.0001*
	7 days	0.780	0.136	3.808	< 0.0001*

*Significant reliability as *P* ≤ 0.05.

### Statistical analysis

Statistical analysis was performed with SPSS 16 ^®^ (Statistical Package for Scientific Studies), Graph pad prism & windows excel and presented in 3 tables and 3 graphs. Exploration of the given data was performed using Shapiro-Wilk test and Kolmogorov-Smirnov test for normality which revealed that data originated from non-parametric distribution. Accordingly, comparison between 2 different groups was performed by Mann Whiteny`s test, while comparison between after 24 h and after 7 days was performed by Wilcoxon signed rank test. On the other hand, regarding qualitative data, all comparisons were performed by using the Chi square test. The significance level was set at *p* ≤ 0.05.

#### Evaluation of pain

##### Intragroup comparison

**Fig. 2:**
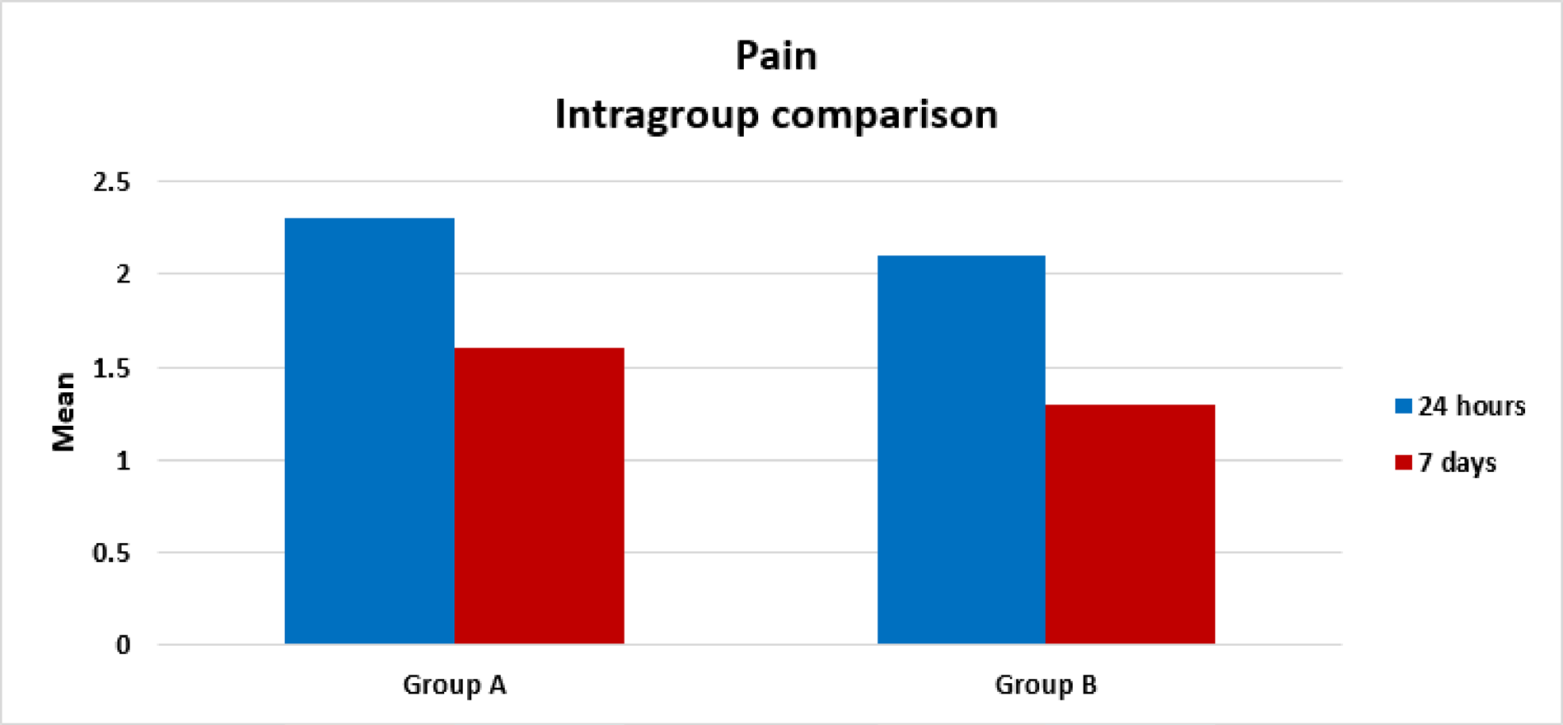
Bar chart showing the Mean values of Pain scores after 24 h and after 7 days in group A (ProTaper Universal System) and group B (M-Pro system) (Intragroup comparison).

There was significant change in pain in both groups. In group A (ProTaper Universal System), there was a significant decrease in pain from (2.3 ± 0.97) after 24 h to (1.6 ± 0.81) after 7 days as *P* = 0.0001. In group B (M-Pro System), there was a significant decrease in pain from (2.1 ± 0.93) after 24 h to (1.3 ± 0.52) after 7 days as *P* = 0.0001, as presented in (Table 2 and Fig. 2).

**Table 2:** Descriptive results of pain in group A and group B, comparison between after 24 h and after 7 days using Wilcoxon signed rank test.

		Minimum	Maximum	Median	Mean	Standard Deviation	*P* value
ProTaper Universal system	24 h	1.00	4.00	2.00	2.30	0.97	0.0001*
	7 days	1.00	4.00	1.00	1.60	0.81	
M-Pro system	24 h	1.00	4.00	2.00	2.10	0.93	0.0001*
	7 days	1.00	3.00	1.00	1.30	0.52	

*Significant difference as *P* < 0.05.

##### Intergroup comparison

**Fig. 3:**
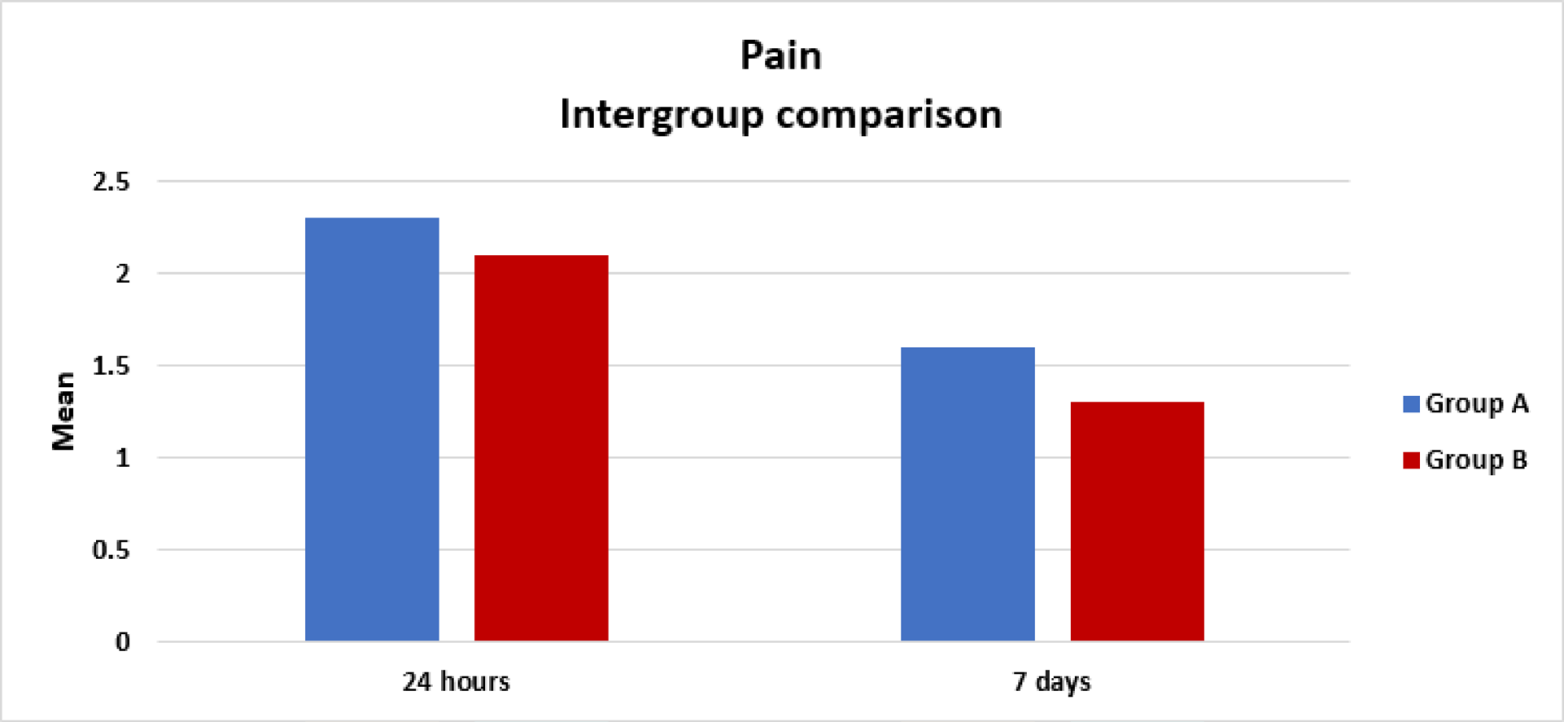
Bar chart showing the Mean values of Pain scores after 24 h and after 7 days in group A (ProTaper Universal System) and group B (M-Pro system) (intergroup comparison).

There was an insignificant difference between group A and group B after 24 h (*P* = 0.38), after 7 days (*P* = 0.07), as showed in (Table 3 and Fig. 3).

**Table 3:** Mean and standard deviation of pain after 24 h and after 7 days, comparison between group A and group B using Mann Whitney’s test.

	ProTaper Universal system		M-Pro system		Mean difference	Std. error difference	95% confidence interval of the difference		*P* value
	M	SD	M	SD			Lower	Upper	
24 h	2.30	0.97	2.10	0.93	0.20	0.21	-0.22	0.62	0.38
7 days	1.60	0.81	1.30	0.52	0.30	0.15	0.00	0.60	0.07

#### Analgesics usage

**Fig. 4:**
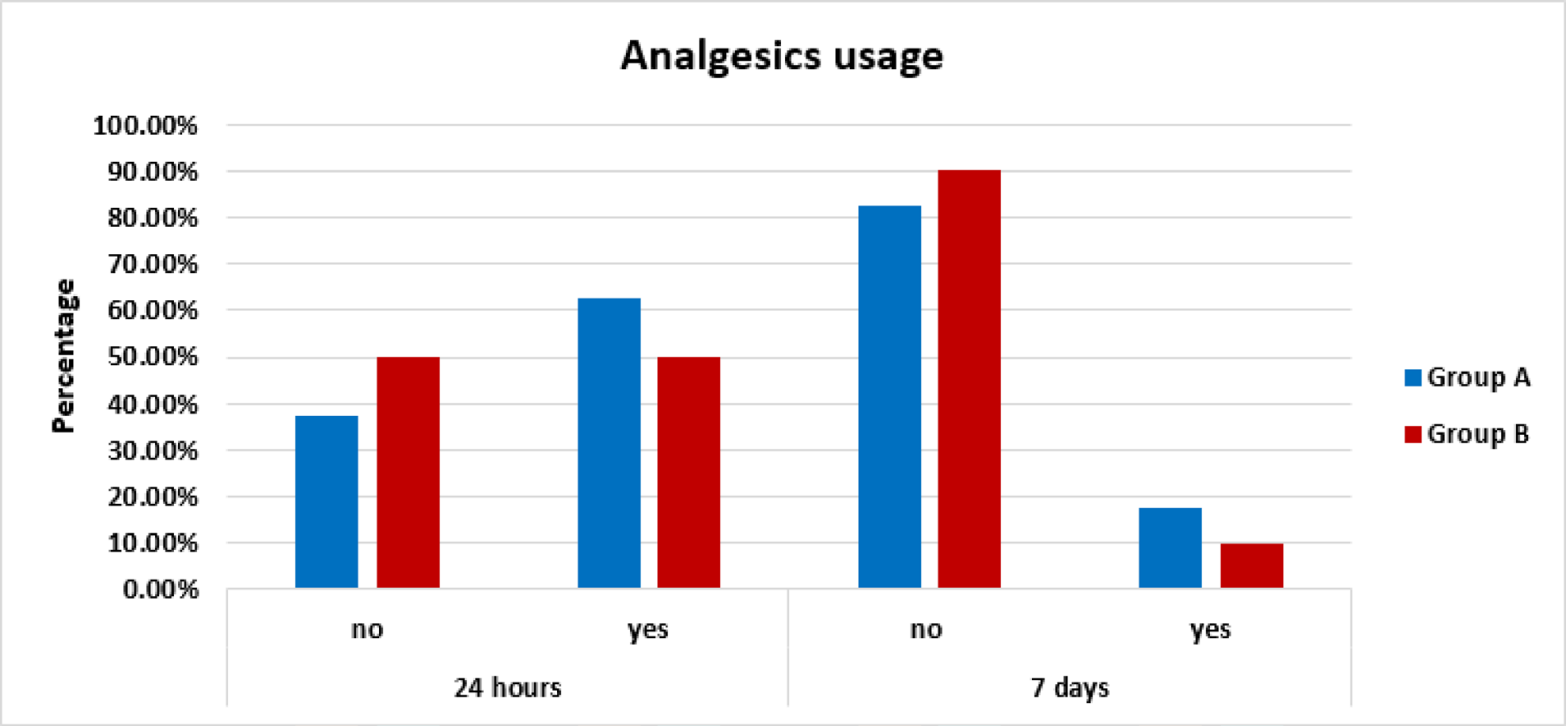
Bar chart showing frequency of analgesics usage among Group A (ProTaper Universal system) and group B (M-Pro system).

Frequency and percentages of analgesics usage in group A and group B after 24 h and after 7 days were shown in (Table 4 and Fig. 4). Chi square test was used for Comparison between them. Chi square test revealed insignificant difference between them as *P* = 0.26, and 0.33 after 24 h and after 7 days respectively.

**Table 4:** Frequency and percentage of analgesic usage in ProTaper Universal system and M-Pro system after 24 h and after 7 days.

Interval	Use analgesics	GroupA ProTaper Universal system		Group B M-Pro system		P value
		Count	Column N %	Count	Column N %	
24 h	No	15	37.5%	20	50.0%	0.26
	Yes	25	62.5%	20	50.0%	
7 days	No	33	82.5%	36	90.0%	0.33
	Yes	7	17.5%	4	10.0%	

## Discussion

Postoperative pain is considered to have a great impact on quality of life; consequently, it is of great importance to control it. Although postoperative pain may occur due to many factors, the instrumentation method is believed to have a major role^22–25^.

Postoperative pain is mostly associated with endodontic treatment of molars more than other tooth types. The complex anatomy of molars makes cleaning and shaping of their canals more difficult, increasing the risk of postoperative complications^26^.

Many studies concluded that using rotary Ni–Ti systems helped in decreasing postoperative pain^27,28^. There are limited clinical studies comparing the pain occurring following the use of different rotary system. Therefore, the objective of this present clinical study was the comparison between the influence of root canal treatment with ProTaper universal and M-Pro endodontic rotary files on the intensity of postoperative endodontic pain.

Metallurgical characteristics of endodontic files have the potential to considerably influence postoperative pain by modulating the extrusion of debris and the flexibility of the instruments. Heat-treated nickel-titanium alloys provide superior flexibility and shape memory, facilitating efficient and less traumatic shaping of root canals, thereby reducing the extrusion of debris and irrigants into the periapical tissues^29^. Heat-treated nickel-titanium files produce reduced torque during operation, which lessens the mechanical stress exerted on the tooth and adjacent tissues, consequently attenuating the factors contributing to postoperative pain^30^.

Debris extrusion loaded by microorganisms may reach the periradicular tissues during root canal treatment causing injury and inflammation of the periapical tissue which promotes secretion of endogenous chemical mediators concurrent with inflammation and pain, as prostaglandins, serotonin, leukotriene, histamine and bradykinin^31,32^.

The MPro system is a relatively novel one in the market with few studies about the assessing of the impact of its use on postoperative pain. The MPro system became well known due to its economic popularity and its new technology in metallurgy; as it is made from specially treated raw X-wire material allowing pre-bending of the files and adding advantages to their stiffness and strength. The manufacturer claims that this will reduce the risk of breakage and will minimize the screw in effect^33^.

In the current study, we have elect ProTaper Universal files to assess the effect of variable file taper on causing post instrumentation pain. According to ProTaper Universal manufacturers, variable file taper reduced torsional loading and file fatigue and improved both flexibility and cutting efficiency. The progressively changing helical angle and pitch helped in achieving instrument balance, reducing the probability of threading and helping in debris removal. The instruments´ short handles is an advantage for easily reaching posterior teeth^34^.

Work Standardization protocol is mandatory to avoid any effects of intraoperative variables on outcomes. In this study, the working length, the type, and volume of irrigant were the same in both groups. In all cases, working length determination was accomplished using an apex locator and radiographs. Side vented needles were used for irrigation to help the irrigation solution to safely reach the apex^35^.

Recently, the single-visit endodontic treatments have become more popular than multiple visits. In our study, single visit root canal treatment was performed consuming lesser time, relatively with less cost, and decreased overall postoperative pain^36^.

Pain is always difficult to study, because it is very depending on the patient evaluation, that´ s why, the methodology used in evaluating pain level is very critical^37^. Many different methods are used to assess pain^24,37^. In our study we used a 10-point NRS because it is easy to use and more sensitive than verbal rating scales (VRSs)^38^. Patients were taught the way to use the scale to assure accurate outcome data assembly^39^.

This clinical study results showed that, there was significant decrease in pain in both groups after 24 h and 7 days. In group A (ProTaper Universal), there was a significant decrease in pain from (2.3 ± 0.97) after 24 h to (1.6 ± 0.81) after 7 days. In group B (M-Pro), there was a significant decrease in pain from (2.1 ± 0.93) after 24 h to (1.3 ± 0.52) after 7 days as, as presented in (Table 1 and Fig. 2).

This was in accordance with Pak and White^1^. The objective of their systematic review was to determine the impact of root canal treatment on the prevalence and intensity of pain, as well as to evaluate the prevalence and severity of pain experienced pretreatment, during treatment, and post treatment. It was observed that the prevalence of pretreatment pain was high, yet it diminished moderately within 1st day and significantly reduced to minimal levels by the 7th day. The severity of pretreatment pain was assessed as moderate, experienced a substantial decline within the first day of treatment, and continued to decrease to minimal levels by the 7th day.

The findings from numerous studies^40–44^ assessing postoperative pain subsequent to endodontic treatment using various file systems across multiple testing time points (such as 3, 6, 12, 24, 48 h, and 7 days) indicated that, a statistically significant difference in postoperative pain was observed solely during the initial 24 h postoperatively, while no statistically significant differences was identified among the various groups during the remaining time intervals.

In light of the aforementioned data, patients instructed to record pain intensity using the NRS at both 24 h and 7 days following the procedure in this study.

Our results revealed that, there was an insignificant difference between group A and group B after 24 h (*P* = 0.38), after 7 days (*P* = 0.07), as shown in (Table 2 and Fig. 3).

This was in agreement with Elnahas et al.^45^. , who found no statistically significant difference related to pain intensity after using ProTaper Next files or Neolix files (controlled memory files) at 6, 12 and 24 h postoperatively.

This results was against that found by Al Morad et al.^40^. , which displayed a statistically significant escalation in the prevalence of postoperative pain within M-Pro group in comparison to TF Adaptive group at 24 h postoperatively, while a statistically insignificant difference was noted between the two groups during subsequent time intervals of 6, 12 and 48 h.

Our results showed that, at the two different follow-up time intervals, the ProTaper and M-Pro groups recorded similar postoperative pain (NRS scores) and analgesic intake.

Comparable findings were revealed by Othman et al.^42^. Their investigation demonstrated statistically insignificant difference between M-Pro group and ProTaper Next group regarding pain NRS scores or analgesic intake at 6, 12, 24, 48 and 72 h postoperatively.

Based on these results, our clinical study found that no difference was found between both systems regarding postoperative pain in molars root canals preparation.

## Conclusions

Postoperative pain was nearly similar, when root canal treatment was performed with either the ProTaper or M-Pro rotary instruments after 24 h and 7 days. No difference in frequency of analgesics intake was noted between both groups. M-Pro endodontic instrument is a promising and more economical instrument for root canal preparation.

### Study limitations

The results need to be confirmed by additional clinical studies, as there may be a difference in study design, patient’s allocation technique and / or methodology of other studies and our study.

### Recommendation

Reducing the discomfort to the patients following root canal treatment is a main target of operators. Further in-vivo studies are required in order to evaluate effect of instrumentation techniques on the incidence and intensity of postoperative pain.

## Data Availability

The data that supports this study’s findings are available from the authors upon reasonable request.
